# Type II restriction endonucleases — a historical perspective and more

**DOI:** 10.1093/nar/gkw513

**Published:** 2016-06-07

**Authors:** Alfred Pingoud, Geoffrey G. Wilson, Wolfgang Wende

**Affiliations:** 1 Institute of Biochemistry, Justus-Liebig-University Giessen, Heinrich-Buff-Ring 58, D-35392 Giessen, Germany; 2 New England Biolabs Inc., 240 County Road, Ipswich, MA 01938–2723, USA

*Nucl. Acids Res*. 42 (12): 7489–7527. doi: 10.1093/nar/gku447

The authors wish to correct reference 228 from:

228. Naito T., Kobayashi I. Cell death programmed by selfish genes–or, why are there restriction enzymes. Jikken Igaku 1995;13:1444–1447.

To

228. Naito T., Kusano K., Kobayashi I. Selfish behavior of restriction-modification systems. Science 1995;267:897–899.

